# Using Yeast Synthetic Lethality To Inform Drug Combination for Malaria

**DOI:** 10.1128/AAC.01533-17

**Published:** 2018-03-27

**Authors:** Suvitha Subramaniam, Christoph D. Schmid, Xue Li Guan, Pascal Mäser

**Affiliations:** a Swiss Tropical and Public Health Institute, Basel, Switzerland; b University of Basel, Basel, Switzerland; c Swiss Institute of Bioinformatics, Basel, Switzerland; d Nanyang Technological University, Singapore

**Keywords:** antimalarials, combinatorial chemotherapy, gene orthology, synthetic lethality, yeast genetics

## Abstract

Combinatorial chemotherapy is necessary for the treatment of malaria. However, finding a suitable partner drug for a new candidate is challenging. Here we develop an algorithm that identifies all of the gene pairs of Plasmodium falciparum that possess orthologues in yeast that have a synthetic lethal interaction but are absent in humans. This suggests new options for drug combinations, particularly for inhibitors of targets such as P. falciparum calcineurin, cation ATPase 4, or phosphatidylinositol 4-kinase.

## TEXT

There is a persistent need for new antimalarials due to the evolution of drug-resistant parasites. Under the auspices of the Medicines for Malaria Venture (MMV), new drug candidates that are active against artemisinin-resistant isolates of Plasmodium falciparum are being developed; the frontrunners are artefenomel, KAF156, cipargamin, DSM265, MMV390048, ferroquine, and tafenoquine (1, 2). However, the choice of the right partner drug will be critical for the success of these new molecules, as the WHO enforces the application of antimalarials in combination therapy (3). In addition to protecting each other from drug resistance, two molecules to be combined need to be compatible for coformulation, should have matching pharmacokinetic profiles, and must not have unfavorable polypharmacology (4–6). Ideally, the two molecules would potentiate each other, thereby decreasing the duration of treatment and the required doses. Thus, combinatorial chemotherapy not only can reduce the risk of drug resistance but also can enhance drug safety and drug efficacy, enabling the ambitious goal of a “single-exposure radical cure” (7, 8).

Here we propose to support the matchmaking of antimalarial candidates by learning from yeast reverse genetics. Saccharomyces cerevisiae is probably the best studied of all eukaryotes. Only about 20% of its protein-coding genes are essential for growth on rich medium (9). High-throughput crossing experiments have shown that many viable S. cerevisiae gene deletion mutants possess synthetic phenotypes, i.e., growth defects that become apparent only in the absence of another nonessential gene. The concept of genetic synthetic lethality can be adopted to combination chemotherapy (8, 10–12). The principal idea is to extrapolate from synthetic lethal gene pairs in S. cerevisiae to orthologous pairs of genes in P. falciparum, assuming that the combined inhibition of the respective gene products will produce a synergistic effect. However, this seemingly straightforward approach is complicated by the fact that S. cerevisiae is more closely related to Homo sapiens than to P. falciparum (13). Thus, a drug combination inferred from yeast synthetic genetic lethality might enhance the toxicity to humans rather than enhancing the antimalarial efficacy. To avoid such a scenario, we developed an algorithm to exclude gene pairs that are conserved in H. sapiens.

**FIG 1:**
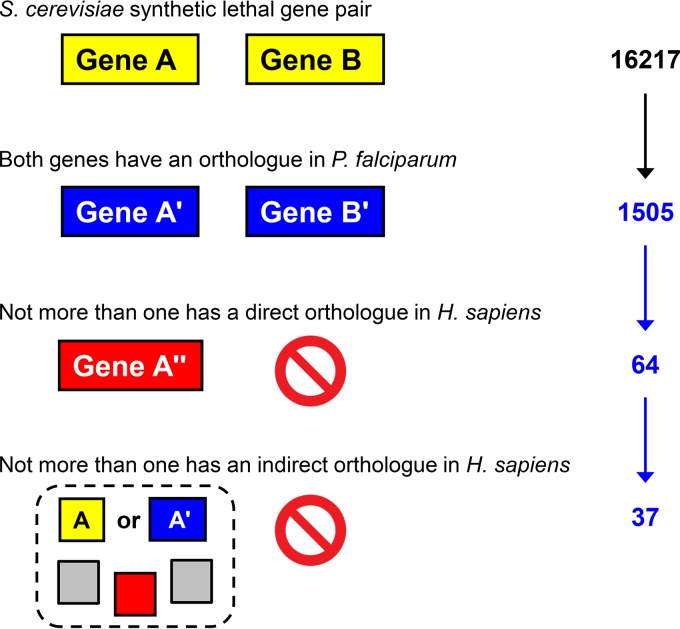
Graphic representation of the algorithm, with the numbers of P. falciparum gene pairs that passed the filters; the final 37 are shown in Table 1. Yellow, S. cerevisiae; blue, P. falciparum; red, H. sapiens.

Yeast synthetic lethal gene pairs were obtained from BioGRID 3.4 (14) and pairs and groups of orthologous genes from the OrthoMCL 5 database, based on the similarity of the derived protein sequences (15, 16). Mining the OrthoMCL database with the 16,217 synthetic lethal gene pairs of S. cerevisiae identified in BioGRID, we found that only 1,505 pairs (9.3%) had direct orthologues in P. falciparum for both gene products (Fig. 1). From this set, we tested all of the proteins for the presence of an orthologue in the human proteome, again referring to the downloaded OrthoMCL database. This assessment included direct pairwise orthology between the P. falciparum or S. cerevisiae protein and a H. sapiens protein or indirect orthology in which either the malaria protein or its yeast orthologue belonged to an OrthoMCL group that also contained a human protein (Fig. 1). All of the P. falciparum gene pairs for which both gene products tested positive for direct or indirect human orthology were eliminated. This process yielded 37 pairs composed of 55 unique P. falciparum proteins that fulfilled the conditions that (i) their direct orthologues in S. cerevisiae exhibit synthetic lethality and (ii) at least one of the two proteins has neither a direct nor an indirect orthologue in the human proteome. Therefore, we suggest these pairs as targets for combinatorial chemotherapy. The comparative genomics pipeline (Fig. 1) is built with self-developed Python scripts that are available for download at the GitHub repository (https://github.com/suvi-subra/SynthLeth).

The final set of 37 pairs was enriched in druggable proteins (Table 1). Of the 55 proteins in the set, 30 either had been validated as drug targets or had a positive “druggability index,” as predicted by TDR Targets (17). Some of the suggested combinations affected the same pathway, e.g., the pyridoxal kinase-like protein and pyridoxine biosynthesis protein involved in vitamin B_6_ metabolism or NAD(P)H-dependent glutamate synthase and NADP-specific glutamate dehydrogenase; the latter is selectively inhibited by isophthalic acid (18), while glutamate synthase had been suggested as a target based on comparative genomics (19). Calcineurin subunit B paired with the P. falciparum cation/H^+^ antiporter (PfCHA), which is sensitive to known inhibitors such as KB-R7943 (20). Hubs of inferred interactions were P. falciparum apurinic/apyrimidinic endonuclease 1 (PfAPN1) and the P. falciparum U5 small nuclear ribonucleoprotein (PfSNU114) of the spliceosome, both of which are involved in the processing of nucleic acids. Two proteins in the target set were of particular pharmacological interest, namely, P. falciparum Ca^2+^-ATPase 4 (PfATP4) and P. falciparum phosphatidylinositol 4-kinase (PfPI4K). Either protein is targeted by new antimalarial candidates (21–27). PfATP4 is the target of cipargamin and paired with PfCHA (Table 1), suggesting testing for potential synergy between cipargamin and KB-R7943. PfPI4K, the target of imidazolopiperazines and MMV390048, paired with ubiquitin-conjugating enzyme E2 (Table 1). An inhibitor of Atg8-Atg3 interactions was identified from the MMV Malaria Box (28), and ubiquitin-protein ligase E3 was proposed as an antimalarial target (29). The inferred link between phosphatidylinositol 4-kinase and ubiquitination suggests testing for potential synergy between PfPI4K inhibitors and P. falciparum proteasome inhibitors (30–32).

**TABLE 1:** Pairs of P. falciparum proteins suggested as targets for combinatorial chemotherapy, based on synthetic lethal genetic interactions in S. cerevisiae

Gene 1 identification	Gene 1 product^*a*^	Gene 2 identification	Gene 2 product
PF14_0492	Calcineurin subunit B	PFF0170w	Cation/H+ antiporter
PFL0590c	Non-SERCA-type Ca^2+^-transporting P-ATPase 4	PFF0170w	Cation/H+ antiporter
PFE0485w	Phosphatidylinositol 4-kinase	PFF0305c	Ubiquitin-conjugating enzyme E2
PF08_0031	Dicarboxylate/tricarboxylate carrier	mal_mito_2	Cytochrome *c* oxidase subunit 1
PFF1105c	Chorismate synthase	PF14_0511	Glucose-6-phosphate dehydrogenase
PFL2465c	Thymidylate kinase	PF13_0176	Apurinic/apyrimidinic endonuclease
MAL13P1.346	DNA repair endonuclease	PF13_0176	Apurinic/apyrimidinic endonuclease
PFB0160w	ERCC1 nucleotide excision repair protein	PF13_0176	Apurinic/apyrimidinic endonuclease
PFF0715c	Endonuclease III homologue	PF13_0176	Apurinic/apyrimidinic endonuclease
PFD0865c	Cdc2-related protein kinase 1	PFF0165c	Conserved Plasmodium protein, unknown function
PFL1635w	Sentrin-specific protease 1	PF10_0092	Metallopeptidase
PF13_0251	DNA topoisomerase 3	PF10_0092	Metallopeptidase
PFF0775w	Pyridoxal kinase-like protein	PFF1025c	Pyridoxine biosynthesis protein
PF11_0192	Histone acetyltransferase	PFF1180w	Anaphase-promoting complex subunit
PFL2440w	DNA repair protein	MAL7P1.94	Prefoldin subunit 3
PF11_0087	DNA repair protein	PF10_0041	U5 small nuclear ribonucleoprotein
PFB0445c	ATP-dependent RNA helicase	PF10_0041	U5 small nuclear ribonucleoprotein
PFE0925c	ATP-dependent RNA helicase	PF10_0041	U5 small nuclear ribonucleoprotein
PF10_0294	Pre-mRNA-splicing factor ATP-dependent RNA helicase	PF10_0041	U5 small nuclear ribonucleoprotein
PFC1060c	U4/U6.U5 tri-small-nuclear-ribonucleoprotein-associated protein 1	PF10_0041	U5 small nuclear ribonucleoprotein
PF13_0096	U4/U6.U5 tri-small-nuclear-ribonucleoprotein-associated protein 2	PF10_0041	U5 small nuclear ribonucleoprotein
PFC0365w	Pre-mRNA-processing factor 19	PF10_0041	U5 small nuclear ribonucleoprotein
PFD0685c	Structural maintenance of chromosomes protein 3	PF10_0041	U5 small nuclear ribonucleoprotein
MAL13P1.214	Phosphoethanolamine *N*-methyltransferase	PFA0455c	Fatty acid elongation protein, GNS1/SUR4 family
MAL13P1.214	Phosphoethanolamine *N*-methyltransferase	PFL0950c	Aminophospholipid-transporting P-ATPase
MAL8P1.17	Protein disulfide isomerase	PF10_0092	Metallopeptidase
MAL8P1.17	Protein disulfide isomerase	PFB0920w	DnaJ protein
PF07_0029	Heat shock protein 86	MAL13P1.139	Mitochondrial fission 1 protein
PF07_0029	Heat shock protein 86	PFI0300w	Vacuolar protein sorting-associated protein 46
PF14_0068	rRNA 2′-*O*-methyltransferase fibrillarin	PFF1180w	Anaphase-promoting complex subunit
PF14_0261	Proliferation-associated protein 2g4	PF14_0612	Zinc finger protein
PF14_0286	NADP-specific glutamate dehydrogenase	PF14_0334	NAD(P)H-dependent glutamate synthase
PF14_0401	tRNA import protein	PF13_0257	Glutamate-tRNA ligase
PFC0510w	E3 ubiquitin-protein ligase	PFI0300w	Vacuolar protein sorting-associated protein 46
PFE0750c	Pre-mRNA-splicing factor	PF14_0688	Pre-mRNA-splicing factor ISY1
PFF1385c	Conserved Plasmodium protein	PFB0920w	DnaJ protein
PFL1140w	Vacuolar iron transporter	PFL0725w	Thioredoxin peroxidase 2

a SERCA, sarcoendoplasmic reticulum calcium transport ATPase; ERCC1, excision repair cross-complementation group 1.

The present approach critically depends on the existence of S. cerevisiae genes that (i) possess synthetic lethal phenotypes and (ii) are orthologous to known P. falciparum drug target genes. This seems contradictory; by definition, drug targets are essential and genes with synthetic phenotypes are nonessential. However, we show here that several validated drug targets of P. falciparum possess orthologues in S. cerevisiae that are nonessential (Table 1). Phosphoethanolamine methyltransferase and phosphatidylinositol 4-kinase (Table 1), for instance, have been demonstrated to be essential enzymes in P. falciparum (24, 33). Most of the genes that are conserved between S. cerevisiae and P. falciparum also have an orthologue in H. sapiens (the OrthoMCL database contains only 80 yeast genes with an orthologue in P. falciparum but not in H. sapiens). We speculate that, of the conserved genes that are essential in yeast, many may also be essential in H. sapiens and their products not suitable as drug targets. On the other hand, conserved genes that are devoid of synthetic phenotypes in yeast might also be disposable in P. falciparum and thus not suitable either. The conserved genes that have synthetic lethal phenotypes in yeast might be the most interesting pharmacologically.

The proposed algorithm strongly narrows the target space for antimalarial drug combinations by including potentially synergistic interactions involving efficacy against P. falciparum as well as toxicity against H. sapiens. The fact that it relies on genome-scale experimental data from S. cerevisiae rather than P. falciparum makes the algorithm straightforward and unbiased but also difficult to validate experimentally. Presently, experimental testing of the identified target pairs in Table 1 is precluded by the lack of inhibitors for most of the proposed targets. However, we think that the presence of targets such as PfPI4K and PfATP4 in Table 1 validates the algorithm, and we hope that the algorithm will help identify future combinations of antimalarial molecules that potentiate each other.
